# Nurses and physicians’ viewpoints about decision making of do not attempt resuscitation (DNAR)

**DOI:** 10.1186/s40248-018-0133-8

**Published:** 2018-07-15

**Authors:** Masoud Fallahi, Somaye Mahdavikian, Alireza Abdi, Fariba Borhani, Parvin Taghizadeh, Behzad Hematpoor

**Affiliations:** 1 Nursing Department, Imam Reza Hospital, Kermanshah University of Medical Sciences, Kermanshah, Iran; 2 Nursing and midwifery school, Kermanshah University of medical sciences, Kermanshah, Iran; 3 Nursing and midwifery school, student research committee, Kermanshah University of Medical Sciences, Kermanshah, Iran; 4 Nursing, medical ethics and law research center, medical surgical nursing department, School of Nursing and Midwifery, Shahid Beheshti University of Medical Sciences, Tehran, Iran; 5 Critical care nursing, Imam Reza hospital, Kermanshah University of Medical Sciences, Kermanshah, Iran; 6 Nursing and midwifery school, Kermanshah University of Medical Sciences, Kermanshah, Iran

**Keywords:** Physician, Nurse, Cardiopulmonary resuscitation

## Abstract

**Background:**

Despite advances with regard to “do not attempt resuscitation order”, physicians are still reluctant to implement it. In fact, while the nurses could be of great help in making decision about “do not attempt resuscitation order,” they are mostly neglected in this process. The current study was conducted to determine the nurses and physicians’ viewpoints about decision making process of “do not attempt resuscitation order”.

**Methods:**

A descriptive analytical study was carried out with participation of 152 physicians and 152 nurses. The participants were selected through stratified quota sampling from three educational hospitals affiliated with Kermanshah University of Medical Sciences. Data gathering tools were a demographics checklist and a researcher-designed questionnaire with 20 statements for measuring the attitudes of the respondents about the decision-making process and implementation of “do not attempt resuscitation order” of incurable patients.

**Results:**

Totally, 304 respondents (152 nurses and 152 physicians) participated in the study. The nurses’ attitude score about the consent of the competent patients to “do not attempt resuscitation” was significantly lower in comparison with the physicians, (*p* < 0.001). However, the nurses’ attitude was more positive than the physicians attitude about the belief that “taking the patient’s consent is the physician’s responsibility” (*p* < 0.001). Moreover, the nurses’ attitude was more negative compared with the physicians’ attitude about the idea that “obtaining the patient’s consent is the nurse’s responsibility” (*p* < 0.001). Both groups believed that the nurses cannot recommend “do not attempt resuscitation order” (*p* < 0.770). Both groups of the respondents believed that the nurses were not qualified to issue the “do not attempt resuscitation order” (physicians’ mean score = 2.85, nurses’ mean score = 2.89). The physicians’ believe in “necessity to negotiate with the nurses about the order” was less deep than that of the nurses (*p* = 0.035).

**Conclusions:**

Given the different attitudes of the nurses and the physicians about the decision-making process of “do not attempt resuscitation,” it is necessary to codify a medical guideline and clarify the decision making and implementation process. The guideline needs to clearly state physician's, nurse's, patient's, and other medical team members’ responsibilities and roles, respectively.

## Background

Cardiopulmonary resuscitation (CPR) was introduced in 1960 and the American Heart Association (AHA) approved it for clinical use in 1974. AHA suggested that if CPR is futile for the patient, forgoing or cutting it is moral [1]. CPR can prevent premature death or on the other hand prolong the suffering and disease of the patient, not to mention the necessity of respecting the patient’s autonomy [2]. Making decision about implementing “do not attempt resuscitation” (DNAR) order is a complicated process that brings several moral and legal concerns [3].

Many physicians have expressed their dissatisfaction with their lack of knowledge of when to issue and implement the order of DNAR and with whom to negotiate. In addition, there is a considerable disagreement about the codification, registration, and implementation of DNAR order [2–4]. Pettersson et al. indicated that the decision for DNAR orders are usually taken too late. Some others are taken exactly on the day of patient’s death. Many doctors believe that these decisions should be taken earlier. Additionally, the findings indicate different opinions about whether or not the patient should be aware of DNAR order [2]. Moreover, nurses are widely challenged by DNAR given the challenging nature of the order from ethical and legal viewpoints. There is a paucity of research works on the nurses’ interactions and experiences with regard to DNAR orders and how they must participate or want to participate in the process of decision making; such orders could be a great source of stress for them [4–7].

DNAR order has been reviewed and used comprehensively in some societies for decades; however, in the face of controversies, there are many differences among different societies in terms of performance, morality, legality, and appropriate medical guidance of DNAR [3–6]. Medical staff consider a variety of factors for making end of life decisions such as: probability of survival, patient’s desire, previous quality of life and anticipated quality of life afterward [8]. Religion is the one of the most important factors that affects the decision making process for DNAR orders. Many Muslims choose CPR without considering the poor prognosis of the disease in the hope that God will ultimately heal the patient. The religious and moral beliefs are the main reasons for not legitimating DNAR orders in the Middle East countries [7].

In Iran there is no a determined law for DNAR order [6]; however, there are evidences that DNAR order is carried out unofficially and without any specific guideline so that there is no specific ethical guideline in this regard [9]. The decisions about DNAR orders are made usually by the physician without documentation, logging, or negotiating the matter with the patients or their family [10]. This trend can lead to negligence of the patient’s right, dignity, and respect. Probably, dignity and respect are the last and most important expectations of the patients in their death bed from the health team and family members [11]. Muslims are the majority in Iran [12] and from the Islamic viewpoint, even a single moment in one’s life counts [10, 12, 13]. Many Muslims reject DNAR order because they believe that it is equal to suicide and lack of faith in God [7, 14]; however, there are legal capacities to legalize DNAR order in Iran by codifying an ethical guideline [15].

There are a few researches comparing attitudes of physicians and nurses toward issuing and implementing DNAR orders in Iran. Therefore, and taking into account the importance of DNAR order for the patients in terminal phase, its challenging nature for the medical staff, lack of routine and legal procedures, and the importance of the medical staff’s attitudes for deciding and executing DNAR order [16], the present study was conducted to compare the nurses and physicians’ attitudes about DNAR order in educational hospitals affiliated with Kermanshah University of Medical Science.

## Methods

### Study design

The study was carried out as a descriptive-analytical work from January 2014 to February 2015. The study population comprised the nurses and physicians working in the hospitals affiliated with Kermanshah University of Medical Science. The sample group consisted of 152 nurses and 152 physicians who were recruited based on the minimum difference of 10%, maximum variance, level of confidence of 95%, and power of 80%. Inclusion criteria for the nurses and the physicians were working in one of three hospitals including Imam Reza, Imam Ali, and Imam Khomeini hospitals and consent to participate in the study. Exclusion criterion was failure to fill out the questionnaires completely. The study protocol was approved by both schools of nursing and midwifery of Shahid Beheshti University of Medical Sciences and Kermanshah University of Medical Sciences (KUMS).

### Study protocol

After approval of the research plan by the Research Department of KUMS, the authors attended the health centers and obtained consent from the heads of the hospitals, heads of nursing offices, and head-nurses of different wards. The participants were selected through stratified-quota sampling after briefing the candidates about the methodology and objectives of the study. The authors randomly visited the wards three times and at three different work shifts and distributed the questionnaires. The participants were ensured about confidentiality and anonymity of their personal information.

The data gathering tools included a demographic checklist and a researcher designed questionnaire about DNAR order. The demographic characteristics included age, gender, job, work experience, work place (hospital), and education. The second questionnaire consisted of 20 statements on the process of issuing and implementing DNAR order including the patient’s consent, the physicians and nurse’s role, responsibility of the physician and the nurse with regard to the process of issuing DNAR order, and implementation of the order. The questionnaire was designed on the basis of literature reviews [4, 5, 8, 17] and consulting ten experts of nursing and medical ethics in Kermanshah and Shahid Beheshti universities of medical sciences. The statements were designed based on Likert’s five-point scale (completely agree = 5, to completely disagree = 1); so that, the higher the score, the more positive the attitude. The statements were interpreted and compared independently. It is notable that the questionnaire was in Farsi.

To test content and face validity, the questionnaire was given to 10 faculty board members in the School of Nursing and Midwifery- Shahid Beheshti, experts of medicine ethics in Shahid Beheshti Medicine Ethics Research Center, and specialists of intensive care in KUMS. Afterward, their comments were implemented on the questionnaire. Reliability of the questionnaire was supported based on Cronbach’s alpha (α = 0.884).

### Data analysis

The collected data was analyzed in SPSS (v.21) using descriptive and inferential statistics. To check normal distribution of the data, Kolmogrov-Smirnov test was used and to determine the mean score of attitude of the participation, descriptive statistics such as mean score, percentage, and SD were used. Knowing that the data was normally distributed, mean scores of attitudes of the participants regarding DNAR order were compared using independent t-test (*p* < 0.05).

## Results

Out of the 222 physicians, 168 (75%) returned the questionnaires filled out and 16 (7.2%) returned the questionnaires not completely filled out. Totally, 152 (68%) questionnaires were examined. Out of 250 nurses, 183 (73%) returned the questionnaires filled out and 31 (12%) returned the questionnaires not completely filled out. Totally 152 questionnaires (61%) were examined. Overall response rate was 64%. Table 1 lists the demographic characteristics of the subjects.

**Table 1:** Demographic characteristics of the subjects

Characteristic		Groups	Frequency (%)
Sex	Nurse	Male	41(27)
		Female	111(73)
	Physician	Male	88(57.8)
		Female	64 (42.2)
Hospital workplace	Nurse	Imam Reza	96(63.2)
		Imam Ali	42(27.6)
		Imam Khomeini	14(9.2)
	Physician	Imam Reza	89(58.6)
		Imam Ali	39(25.6)
		Imam Khomeini	24(15.8)
Age (Year)	Nurse	20-30	67(44.0)
		31-40	59(38.6)
		41–50	23(15.4)
		> 50	3(2.0)
	Physician	20-30	37(24.4)
		31-40	74(48.6)
		4 1-50	30(19.8)
		< 50	11(7.2)
Job experience (Year)	Nurse	1–10	95(62.6)
		11-20	36(23.6)
		21-30	18(11.8)
		> 30	3(7)
	Physician	1-10	96(63.2)
		11-20	28(18.4)
		21-30	21(13.8)
		< 30	7(4.6)
Educational qualification	Nurse	Bachelor	140(92.0)
		MSC	12(7.8)
	Physician	General Physician	31 (20.4)
		Resident	71(46.6)
		Specialist	36 (23.6)
		Physician Professor	14 (9.2)

The nurses’ attitudes score toward obtaining the consent of competent patients was lower than that of the physicians (*p* < 0.001). In addition, the former group’s attitude about the idea that “securing the patient’s consent is the physicians responsibility” was more positive than that of the latter (*p* < 0.001). The nurses’ attitudes about the idea that taking the patient’s consent is the nurses’ responsibility took less score than the physicians. The findings indicated that with regard to the issues that the physicians and the nurses might have different ideas about, both groups responded that “the nurses must follow the physician’s order” (means score of physicians = 3.46, mean score of the nurses = 3.49). Both groups believed that “nurses have to perform DNAR order even when they do not agree with the physician”; while the physicians had stronger attitudes in this regard (*p* < 0.001). Both groups believed that “nurses cannot recommend DNAR order” (mean score of physicians = 3.54; mean score of the nurses = 3.24). Both groups believed that DNAR order is under physician’s authority (mean score of physicians = 3.54; mean score of nurses = 3.24). However, the participants argued that the order must be issued after consulting other physicians, ethics committee, and nurses. There was a significant difference between the nurses and physicians about the “nurses’ role in the process of issuing and implementing DNAR order” so that the latter group’s support of the idea of consulting the nurses about DNAR order was less than that of the former (*p* = 0.035) (Table 2).

**Table 2:** Questionnaire of attitudes about DNAR order

Questionnaire of attitudes about DNAR order		Mean	Median	SD	t	Probability
1. Obtaining the competent patient’s consent for DNAR order is essential	nurse	3.28	4	1.197	3.788	*P < 0.001
	physician	3.77	4	1.070		
2. Obtaining consent of the competent patient’s family is essential for DNAR order.	Nurse	3.53	4	1.156	−1.340	*P* = 0.181
	Physician	3.70	4	1.067		
3. Obtaining consent of incompetent patient’s family is essential for DNAR order.	Nurse	3.05	4	1.141	−1.032	*P* = 0.303
	Physician	3.18	3	1.193		
4. If a competent patient’s family prefers not to inform its patient about DNAR order, the request must be respected.	Nurse	3.06	3	1.111	2.476	*P = 0.014
	Physician	2.74	4	1.113		
5. If a competent patient desires that his/her family shall not be informed about DNAR order, his/her request must be respected.	Nurse	3.45	4	1.078	0.532	*P* = 0.595
	Physician	3.38	4	1.079		
6. The DNAR status of patients must be determined before hospitalization.	Nurse	3.37	3	1.155	0.495	*P* = 0.621
	Physician	3.30	3	1.162		
7. Preferences of the patient about DNAR order must be taken in advance for the patients who may lose their competence.	Nurse	3.32	4	1.040	−0.328	*P* = 0.743
	Physician	3.36	4	1.058		
8. The CPR of patient is immoral if the patient’s family has not given the consent for DNAR order.	Nurse	3.56	4	1.096	−1.387	*P* = 0.166
	Physician	3.73	4	1.026		
9. Obtaining the patient/family’s consent for DNAR order is the physician’s responsibility.	Nurse	3.82	2	1.036	3.710	*P < 0.001
	Physician	3.37	3	1.095		
10. Obtaining the patient/family’s consent for DNAR order is the nurse’s responsibility.	Nurse	2.37	4	1.021	−3.825	*P < 0.001
	Physician	2.83	4	1.106		
11. Obtaining the patient/family’s consent for DNAR order is joint responsibility of the physician and nurse.	Nurse	3.38	3	1.150	−1.457	*P* = 0.146
	Physician	3.56	3	0.968		
12. Upon the patient/family’s consent about DNAR order, the physician must not attempt CPR even if he is not sure about futility of CPR.	nurse	3.01	4	1.171	1.211	*P* = 0.227
	physician	2.83	4	1.289		
13. The nurse should follow the physician’s order for doing CPR even if it is against the patient/family’s desire.	Nurse	3.49	4	1.185	0.240	*P* = 0.810
	Physician	3.46	4	1.201		
14. The nurses shall follow the DNAR order even if they do not agree with order.	nurse	3.26	3	1.096	3.80	*P < 0.001
	physician	3.73	4	1.042		
15. The nurse can recommend DNAR order.	Nurse	2.89	3	1.174	0.292	*P* = 0.770
	Physician	2.85	3	1.181		
16. The attending physician shall make decision about DNAR order.	Nurse	3.24	3	1.151	3.151	*P* = .621
	Physician	3.54	3	1.168		
17. DNAR order should be taken by the physician after consulting ethics committee of the hospital.	Nurse	3.57	4	1.033	0.577	*P* = 0.564
	Physician	3.64	3	0.953		
18. DNAR order must be taken by the physician after consulting with the nurse.	Nurse	3.31	3	1.006	2.122	*P = 0.035
	Physician	3.08	3	0.939		
19. Making decision about DNAR order by the attending physician must be after consulting with other physicians.	Nurse	3.74	3	0.952	0.947	*P* = 0.344
	Physician	3.84	3	0.862		
20. Making decision on DNAR order must be done by ethics committee of the hospital.	Nurse	2.83	3	1.150	0.937	*P* = 0.350
	Physician	2.71	4	1.052		

1-CPR(Cardio Pulmonary Circulation) 2- DANR(Do Not Attempt Resuscitation)

*is significant

## Discussion

The findings showed that doctors and nurses have disagreements in some areas of decision-making process about DNAR order such as the nurses’ role in the decision-making process and obtaining consent from families and patients. However, the physicians‘ attitudes were more positive than the nurses’ attitude about obtaining consent of the patient with the ability to make decision about DNAR. Hosaka et al. showed that the attitudes of 35% of the nurses were more positive comparing with 14% of the physicians with regard to obtaining the patient’s consent about DNAR order [17] that it is different from our study, in which physicians have more positive attitude about patients` consent to DNAR. Ghajarzadeh et al. reported that mostly the doctors proposed DNAR [18]. Yang et al. found that the physicians believed that they were the only authority to undertake DNAR decision [19]. Park et al. in line with our study, showed that the majority of the nurses believed that the patients and their family need to take part in the decision making process by keeping a close and continuous communication with the medical team [20].

The nurses’ attitudes about the idea that obtaining the patients’ consent is the physician’s responsibility were more positive than the physicians’ attitudes; while the former’s attitudes were more negative, comparing with the latter, about the idea that obtaining the patients’ consent is the nurses’ responsibility. In the Moghaddaysian et al. study (2014) the majority of nurses stated that talking with patient and his family about DNR order is difficult [21]. Consistently, O’hanlon et al. showed that the nurses believed that obtaining the patient’s consent about DNAR decision was the physicians’ responsibility; while, only 25% of the nurses expressed that obtaining the patient’s consent was the nurses’ responsibility [4]. Loofmark reported that obtaining the patient’s consent to DNAR order was the physician’s responsibility [22]. Inconsistently with our results and those noted above, Sulmasy et al. reported that the nurses’ attitudes were more positive, comparing with the physicians’ attitudes, about negotiating with the patient and the family members about DNAR order [23]. It seems that low self-confidence, lack to trust in the physicians’ knowledge, ambiguous legal process and the laws about DNAR order in Iran explain the negative attitudes of the nurses about their liability to obtain the patient’s consent about DNAR order.

The physicians believed that they should order DNAR when they find that resuscitation is useless even if the patient and their family disagree. The principle of patient’s autonomy states that the patients might have different ideas from the physician’s about the proper treatment, and the physician shall respect the patient’s autonomy. However, respecting the patient’s independence must not be only for the sake of practicing autonomy and the physicians should make decision based on their knowledge and interests of the patient. That is, if the physician does not agree with the patient, he should give his place to another physician [9, 24].

Both groups of the participants believed that even when the nurses disagree with DNAR order, they should follow the physicians’ order; while the physicians’ attitudes in this regard were more positive. This finding is consistent with the result by O’hanlon et al. [4]. Rakas showed that the physicians would discuss the matter with the nurses when they find out that the nurse disagrees with DNAR order; so that the physicians would give health care program after negotiating it with nurses. The nurses expressed that they would discuss their concern with the physician and ask the reasons for medical decisions. They also answered that since the nurse is the defender of the patient’s rights, they would discuss the terms with the patients and their family and report the case to the manager; however they would never disobey the physician [3].

In addition to the physicians, the nurses believed that they cannot recommend the idea of DNAR. However, Sulmasy et al. mentioned that most of the nurses and the physicians believed that nurses can recommend the subject of DNAR [23]. The inconsistency might be due to the fact that participating nurses in Sulmasy’s study had more active role in providing health services and decision making about the treatments than the nurses in this research.

Both groups believed that it is the physician’s responsibility to give DNAR order, while they expressed the necessity that this must be done after consulting other physicians, ethics committee, and the nurses. In this regard, in Makdasyan study, the nurses believed that the decision to treat the patient was the responsibility of the physician [21]. In the Chakraborty study the doctor is the best person to decide on issues related to the end of life of the patient [25].

Giles and Moule reported that 90% of the nurses believed that the physicians must consult them about the DNAR order regardless of the patient’s ability to make such decision. The nurses also stated that their viewpoints about DNAR were valuable [26]. Abstract highlighted that the nurses believed that DNAR order must be dealt with as a group of decision makers [5]. In 2003 Cardoso et al. in 2003 reported that only 26% of the specialists believed that the nurses can take part in the process of decision making about DNAR order; while more experienced physicians believed the nurses must take part in the decision making process [27]. Nurses could fulfill a key role in the decision making process of DNAR order, as they are a key part of the health care system and provision of health services [28]. In addition, the nurses closely interact with the patients and their family and they must be informed about the key ethical and legal issues of DNAR order [3, 26].

Excluding the nurses from the decision-making process of DNAR [23] is contrary to standards of conduct, performance, and ethics of nursing, which state the nurse acts in the capacity of the attorney of their patients and should protect their interests and rights as much as possible [5, 29]. One may argue that, comparing with the physician, the nurse does not have the knowledge and competence to initiate and lead the ethical concerns of DNAR order. However, this viewpoint is not supported by the findings that indicate the physicians have unacceptable performance regarding DNAR order; as the physicians have expressed their dissatisfaction in this regard [30]. There are also evidences that such cooperation reduces occupational fatigue and burnout in nurses. This indicates the necessity of a coordination and agreement between the attitudes of nurses and physicians [31].

One of the limitations of the present study was its restriction to the three educational hospitals in Kermanshah-Iran. In addition, many of the participants (about 36%) left the study, which may have affected the results. Therefore, the findings must be generalized with cautious. In addition, although attitudes regard the nature of the behavior, knowledge, skills, and behavior toward DNAR order were not measured in the study.

## Conclusion

The findings raised several questions about the physician's and the nurse’s role and responsibility toward the patient in his/her death bed, and also the role of the patients and their family members. Thus, there is a need to improve our understanding of the nurses', patients', and their family’s role in decision making process about the end of life issues including DNAR order. It is also essential to codify a guideline for DNAR order to clearly determine the physicians', the nurses', the patients, and their family members’ role and authority about the order. Although, DNAR order is not legal in Iran, proposing a guideline based on Islamic-Iranian culture could be a step toward legalization of the process and avoiding unofficial and unsupervised implementation of DNAR order.

## Abbreviations

AHA: American Heart Association
CPR: Cardiopulmonary resuscitation
DNAR: do not attempt resuscitation
KUMS: Kermanshah University of Medical Sciences
